# Frames for the Solution of Operator Equations in Hilbert Spaces with Fixed Dual Pairing

**DOI:** 10.1080/01630563.2018.1495232

**Published:** 2018-12-01

**Authors:** Peter Balazs, Helmut Harbrecht

**Affiliations:** a Austrian Academy of Sciences, Acoustics Research Institute, Vienna, Austria;; b Department of Mathematics and Computer Science, University of Basel, Basel, Switzerland

**Keywords:** Banach frames, discretization of operators, frames, invertibility, matrix representation, Stevenson frames

## Abstract

For the solution of operator equations, Stevenson introduced a definition of frames, where a Hilbert space and its dual are *not* identified. This means that the Riesz isomorphism is not used as an identification, which, for example, does not make sense for the Sobolev spaces $H_{0}^{1}(\Omega )$ and $H^{-1}(\Omega )$. In this article, we are going to revisit the concept of Stevenson frames and introduce it for Banach spaces. This is equivalent to $\ell^{2}$-Banach frames. It is known that, if such a system exists, by defining a new inner product and using the Riesz isomorphism, the Banach space is isomorphic to a Hilbert space. In this article, we deal with the contrasting setting, where H and H′ are not identified, and equivalent norms are distinguished, and show that in this setting the investigation of $\ell^{2}$-Banach frames make sense.

## 1. Introduction

The standard definition of frames found first in the paper by Duffin and Schaefer [1] is the following:

(1) $${\left\|f\right\|}_{H}\approx {\left\|{〈f,\psi_{k}〉}_{H}\right\|}_{\ell^{2}}\;\text{for}\;\text{all}\;f\in H.$$

Here, x≈y means that there are constants 0<A≤B<∞ such that A·x≤y≤B·x.

This concept led to a lot of theoretical work, see e.g., [2–6], but has been used also extensively in signal processing [7], quantum mechanics [8], acoustics [9], and various other fields.

Frames can be used also to represent operators. For the numerical solution of operator equations, the (Petrov-) Galerkin scheme [10] is used, where operators are represented by ${〈O\psi_{k},\phi_{l}〉}_{k,l\in K}$, called the *stiffness* or *system matrix*. The collection $\Psi ={\left(\psi_{k}\right)}_{k\in K}$ consists of the ansatz functions, the collection $\Phi ={\left(\phi_{k}\right)}_{k\in K}$ are the test functions. If Ψ and Φ live in the same space, this is called Galerkin scheme, otherwise it is called Petrov–Galerkin scheme.

In finite and boundary element approaches, not only bases were used [11, 12], but also frames have been applied, e.g., in [13–17]. Recently, such operator representations got also a more theoretical treatment [18–20].

In numerical applications, it is often advantageous to have self-adjoint matrices, e.g., for Krylov subspace methods, which necessitates to use the same sequence for the discretization at both sides, i.e., investigating ${〈O\psi_{k},\psi_{l}〉}_{k,l\in K}$. Note that this matrix is self-adjoint if *O* is, and semi-positive if *O* is. Positivity is in general not preserved, only if a system without redundancy is used, i.e., a Riesz sequence. Partial differential operators are typically operators of the form O:H→H′, while boundary integral operators might also be smoothing operators which map in accordance with O:H′→H. One possible solution is to work with Gelfand triples i.e., $H\subset H_{0}\subset H\prime$. This is explicitly done for the concept of Gelfand frames [21].

Another possibility is the following, introduced by Stevenson in [17] and used, e.g., in [15]: A collection $\Psi ={\left(\psi_{k}\right)}_{k\in K}\subset H$ is called a (Stevenson) frame for H, if

(2) $${\left\|f\right\|}_{H\prime }\approx {\left\|{〈f,\psi_{k}〉}_{H\prime ,H}\right\|}_{\ell^{2}}\;\text{for}\;\text{all}\;f\in H\prime .$$

Note the difference to the definition (1) by Duffin and Schaefer, which is significant only if the Riesz isomorphism is not employed. Here, the Gelfand triple is only implicitly used and, if the fully general setting is used, the density of the spaces is not required.

Clearly, the definitions (1) and (2) are equivalent by the Riesz isomorphism. On the other hand, if the isomorphism H≅H′ is not considered, but another one is utilized, for example, considering the triple $H\subset H_{0}\subset H\prime$, then the Riesz isomorphism is usually used as an identification on the pivot space $H_{0}≅H_{0}^{\prime }$, and therefore H and H′ cannot be considered to be equal.

In this article, we consider the original definition by Stevenson and re-investigate in full detail all the derivation to ensure that the Riesz identification does not ‘creep in’ again.

On a more theoretical level, let us consider Banach frames [22–24]. Thus, we consider a Banach space *X*, a sequence space *X_d_*, and a sequence Ψ⊂X′. This is a *X_d_*-frame if

$${\left\|f\right\|}_{X}\approx {\left\|{〈f,\psi_{k}〉}_{X,X\prime }\right\|}_{X_{d}}\;\text{for}\;\text{all}\;f\in X.$$

It is called a Banach frame if a reconstruction operator exists, i.e., there exists $R:X_{d}\to X$ with $R\left(\psi_{k}(f)\right)=f$ for all f∈X.

In this setting, $\ell_{2}$-frames were not considered to be interesting as they are isomorphic to Hilbert frames, see e.g., [25, Proposition 3.10]: Let Ψ be a $\ell^{2}$-frame for X. Then, X can be equipped with an inner product ${〈f,g〉}_{X}={〈C_{\Psi }f,C_{\Psi }g〉}_{\ell^{2}}$, becoming a Hilbert space, and Ψ is a (Hilbert) frame for *X*. The proof uses the Riesz isomorphism H≅H′ in the last line. But if a context is considered, where this isomorphism cannot be applied, like for example a Gelfand triple setting, suddenly the concept of $\ell_{2}$-frames might become nontrivial again, and the concept of Stevenson frames is different to a (standard Hilbert space) frame. In this article, we investigate this approach.

The rest of this article is structured as follows. In Section 2, we motivate Gelfand triples $H\prime \subset H_{0}\subset H$ by a simple example arising from the variational formulation of second-order elliptic partial differential equations. Section 3 then provides the main ingredients we need, especially it introduces the different notions of frames for solving operator equations. By an illustrative example, we show that Stevenson frames seem to offer the most flexible concept for the discretization of operator equations. Finally, in Section 4, we generalize Stevenson frames to Banach spaces and discuss the consequences.

## 2. Motivation: Solving operator equations

Let O:H→H′ and define the bilinear form a:H×H→R by a(u,v)=〈Ou,v〉. Assume that *a* satisfies the following properties:

1. Let *a* be bounded, i.e., there is a constant *C_S_*, such that
  $$a(u,v)\leq C_{S}\cdot {\left\|u\right\|}_{H}{\left\|v\right\|}_{H}.$$
  This is equivalent to *O* being bounded.
2. Let *a* be elliptic, i.e., there exists a constant *C_E_* such that
  $$a(u,u)\geq C_{E}\cdot {\left\|u\right\|}_{H}^{2}.$$
  Both conditions are equivalent to *O* being bounded, boundedly invertible, and positive, see e.g., [26, 27].

The general goal is to find the solution u∈H such that

(3) a(u,v)=ℓ(v) for all v∈H.

This is the weak formulation of the operator equation Ou=b, setting $\ell (v)={〈b,v〉}_{H\prime ,H}$ for u∈H and b∈H′.

In numerical approximation schemes, to get an approximate solution, finite dimensional subspaces V⊂H are considered and the solution $u_{V}\in V$ such that

$$a(u_{V},v)=\ell (v)\;\text{for}\;\text{all}\;v\in V$$

is calculated. The error between the continuous solution u∈H and the approximate solution $u_{V}\in V$ is orthogonal to the space *V*, which is known as the Galerkin orthogonality: $a(u-u_{V},v)=0$ for all v∈V. Note that, in difference to, e.g., a Gelfand triple approach, the norms on *V* and H are the same in the setting above. Instead, the Gelfand triple setting would be $H\subset H_{0}\subset H\prime$ with ${\left\|\cdot \right\|}_{H_{0}}\leq c{\left\|\cdot \right\|}_{H}$.

We shall illustrate the setting also by a practical example from the theory of partial differential equations. To that end, assume that Ω is a bounded domain in $R^{d}$ and let $H_{0}:=L^{2}(\Omega )$ be the space of all square-integrable functions v:Ω→R. As space $H\subset H_{0}$ we consider the Sobolov space $H_{0}^{1}(\Omega )$ which consists of all functions in $L^{2}(\Omega )$ whose first-order week derivatives are also square-integrable and which are zero at the boundary ∂Ω. Thus, the variational formulation of the Poisson equation

−Δu=f in Ω, u=0 on ∂Ω

reads

(4) $$\text{seek}\;u\in H_{0}^{1}(\Omega )\;\text{such}\;\text{that}\;a(u,v)=\ell (v)\;\text{for}\;\text{all}\;v\in H_{0}^{1}(\Omega ),$$

compare [26] for example. The bilinear form

$$a:H_{0}^{1}(\Omega )\times H_{0}^{1}(\Omega )\to R,\;a(u,v)=\int_{\Omega }\nabla u\nabla vdx$$

is continuous and elliptic due to Friedrichs’ inequality, cf. [26], and the linear form

$$\ell :H_{0}^{1}(\Omega )\to R,\;\ell (v)=\int_{\Omega }fvdx$$

is continuous provided that $f\in H^{-1}(\Omega )=(H_{0}^{1}(\Omega ))\prime$. Hereby, the inner product in the pivot space $L^{2}(\Omega )$ is continuously extended onto the duality pairing $H^{-1}(\Omega )\times H_{0}^{1}(\Omega )$. Hence, the underlying Gelfant triple is $H_{0}^{1}(\Omega )\subset L^{2}(\Omega )\subset H^{-1}(\Omega )$.

## 3. Main definitions and notations

### 3.1. Dual pairs

Let *X*, *Y* be vector spaces and *a*(*x*, *y*) a bilinear functional on *X* ×* Y*. Then (*X*, *Y*) is called a dual pair [28], if

1. 

∀x∈X\{0}∃y∈Y s.t. a(x,y)=0,

2. 

∀y∈Y\{0}∃x∈X s.t. a(x,y)=0.

In short, the notation $a(x,y)={〈x,y〉}_{a}=〈x,y〉$ is used. A classical example is a Banach space *X* and its dual space X′. But looking at other dual pairs allows to have an explicit form for the dual elements [29].

Note that often an isomorphism is considered as an identity. For example, by using the Riesz mapping H≅H′, the dual space H′ is often identified with H. If two or more isomorphisms are involved, this identification, of course, can only be considered for one of those isomorphisms. For example, if we consider two Hilbert spaces $H_{1}\subset H_{2}$, the Riesz isomorphism can be considered only for one of them to be an identification, see also Section 3.3.2.

### 3.2. Gelfand triples

Let *X* be a Banach space and H a Hilbert space. Then, the triple (X,H,X′) is called a Banach Gelfand triple [30], if X⊂H⊂X′, where *X* is dense in H, and H is $w^{⋆}$-dense in X′. The prototype of such a triple is $\left(\ell^{1},\ell^{2},\ell^{\infty }\right)$ in case of sequence spaces.

Note that, even if we consider the spaces all being Hilbert spaces – such a sequence is also called rigged Hilbert spaces [31] – the Riesz isomorphism, in general, is not just the composition of the inclusion with its adjoint. This depends on the chosen concrete dual pairing.

As another example, consider the triple $H_{0}^{1}(\Omega )\subset L^{2}(\Omega )\subset H^{-1}(\Omega )$, which has been presented in the practical example for the Poisson equation in Section 2.

### 3.3. Frames

A sequence $\Psi ={\left(\psi_{k}\right)}_{k\in K}$ in a separable Hilbert space H is a *frame* for H, if there exist positive constants $A_{\Psi }$ and $B_{\Psi }$ (called lower and upper frame bound, respectively) that satisfy

(5) $$A_{\Psi }\|f{\|}^{2}\leq \sum_{k\in K}|〈f,\psi_{k}〉{|}^{2}\leq B_{\Psi }\|f{\|}^{2}\;\text{for}\;\text{all}\;f\in H.$$

An upper (resp. lower) semi-frame is a complete system that only satisfies the upper (resp. lower) frame inequality, see [32, 33]. A frame where the two bounds can be chosen to be equal, i.e., $A_{\Psi }=B_{\Psi }$, is called *tight*. We will denote the corresponding sequences in H by $\Psi ={\left(\psi_{k}\right)}_{k\in K}$ and $\Phi ={\left(\phi_{k}\right)}_{k\in K}$ in the following, where we consider general discrete index sets $K\subset R^{d}$. A sequence that is a frame for its closed linear span is called a frame sequence.

By $C_{\Psi }:H\to \ell^{2}$ we denote the *analysis operator* defined by ${\left(C_{\Psi }f\right)}_{k}=〈f,\psi_{k}〉$. The adjoint of $C_{\Psi }$ is the *synthesis operator* $D_{\Psi }(c_{k})=\sum_{k}c_{k}\psi_{k}$. The *frame operator* $S_{\Psi }=D_{\Psi }C_{\Psi }$ can be written as $S_{\Psi }f=\sum_{k}〈f,\psi_{k}〉\psi_{k}$. It is positive and invertible. Note that those ‘frame-related’ operators can be defined as possibly unbounded operators for any sequence in the Hilbert space [34].

By using the *canonical dual frame* $\left({\tilde{\psi }}_{k}\right)$, i.e., ${\tilde{\psi }}_{k}=S_{\Psi }^{-1}\psi_{k}$ for all *k*, we get a reconstruction formula:

$$f=\sum_{k}〈f,\psi_{k}〉{\tilde{\psi }}_{k}=\sum_{k}〈f,{\tilde{\psi }}_{k}〉\psi_{k}\;\text{for}\;\text{all}\;f\in H.$$

The *Gramian matrix* $G_{\Psi }$ is defined by ${\left(G_{\Psi }\right)}_{k,l}=〈\psi_{l},\psi_{k}〉$, also called the mass matrix. This matrix defines an operator on $\ell^{2}$ by matrix multiplication, corresponding to $G_{\Psi }=C_{\Psi }D_{\Psi }$. Similarly, we can define the *cross-Gramian matrix* ${\left(G_{\Psi ,\Phi }\right)}_{k,l}=〈\phi_{l},\psi_{k}〉$ between two different frames Φ and Ψ. Clearly,

$$G_{\Psi ,\Phi }c=\sum_{l}{\left(G_{\Psi ,\Phi }\right)}_{k,l}c_{l}=〈\sum_{l}c_{l}\phi_{l},\psi_{k}〉=C_{\Psi }D_{\Phi }c.$$

If, for the sequence Ψ, there exist constants $A_{\Psi },B_{\Psi }>0$ such that the inequalities

$$A_{\Psi }{\left\|c\right\|}_{2}^{2}\leq {\left\|\sum_{k\in K}c_{k}\psi_{k}\right\|}_{H}^{2}\leq B_{\Psi }{\left\|c\right\|}_{2}^{2}$$

are fulfilled, Ψ is called a *Riesz sequence*. If Ψ is complete, it is called a *Riesz basis*.

#### 3.3.1. Banach frames

The concept of frames can be extended to Banach spaces [22–24]:

Let *X* be a Banach space and *X_d_* be a Banach space of scalar sequences. A sequence $\left(\psi_{k}\right)$ in the dual $X^{\prime }$ is called an *X_d_*-frame for the Banach space *X*, if there exist constants $A_{\Psi },B_{\Psi }>0$ such that

$$A_{\Psi }\|f{\|}_{X}\leq {\left\|\psi_{k}(f)\right\|}_{X_{d}}\leq B_{\Psi }\|f{\|}_{X}\;\text{for}\;\text{all}\;f\in X.$$

An *X_d_*-frame is called a Banach frame with respect to a sequence space *X_d_*, if there exists a bounded reconstruction operator $R:X_{d}\to X$, such that $R\left(\psi_{k}(f)\right)=f$ for all f∈X. In our setting, we use *p*-frames, that is $X_{d}=\ell^{p}$ for 1≤p≤∞, especially, we use $X_{d}=\ell^{2}$.

A family ${\left(g_{k}\right)}_{k\in K}\subset X$ is called a *q-Riesz sequence* (1≤q≤∞) for *X*, if there exist constants $A_{\Psi },B_{\Psi }>0$ such that

(6) $$A_{\Psi }{\left(\sum_{k\in K}|d_{k}{|}^{q}\right)}^{\frac{1}{q}}\leq {\left\|\sum_{k\in K}d_{k}g_{k}\right\|}_{X}\leq B_{\Psi }{\left(\sum_{k\in K}|d_{k}{|}^{q}\right)}^{\frac{1}{q}}$$

for all finite scalar sequence $\left(d_{k}\right)$. The family is called a *q*-Riesz basis if it fulfills (6) and $\bar{\mathrm{span}}\{g_{k}:k\in K\}=X$.

Any *q*-Riesz basis for $X^{\prime }$ is a *p*-frame for *X*, where $\frac{1}{p}+\frac{1}{q}=1$, compare [23].

#### 3.3.2. Gelfand frames

A frame for H is called a *Gelfand frame* [21] for the Gelfand triple (X,H,X′) if there exists a Gelfand triple of sequence spaces $\left(X_{d},\ell^{2},X_{d}^{\prime }\right)$, such that the synthesis operator $D_{\Psi }:X_{d}\to X$ and the analysis operator $C_{\tilde{\Psi }}:X\to X_{d}$ are bounded. As a result, see [21, 35], this means that Ψ is a Banach frame for *X_d_* and $\tilde{\Psi }$ a Banach frame for $X_{d}^{\prime }$.

In many approaches, see e.g. [21], it is assumed for the implementation that there exists an isomorphism $D_{B}:X_{d}\to \ell^{2}$. Should *X_d_* be nonreflexive, then it is also assumed that $D_{B}^{⋆}$ is an isomorphism. If *D_B_* is a diagonal operator, i.e., $D_{B}=\text{diag}\left(w_{k}\right)$ and $D_{B}^{-1}=\text{diag}\left(\frac{1}{w_{k}}\right)$, then $\Psi =(\frac{1}{w_{k}}\psi_{k})$ is a Hilbert frame for *X* and $\left(w_{k}{\tilde{\psi }}_{k}\right)$ is a Hilbert frame for X′. This is shown for real weights in [36]. It is easy to see also for complex weights when using a weighted frame viewpoint [37, 38]. These cases cover the weighted spaces $\ell_{w}^{2}$.

The above setting can be generalized as follows: We define, similar to [25], the sesquilinear form ${〈f,g〉}_{X}^{o}:={〈D_{B}C_{\tilde{\Psi }}f,D_{B}C_{\tilde{\Psi }}g〉}_{\ell^{2}}$. It is obviously bounded and elliptic, and, in particular, ${\left\|f\right\|}_{X^{o}}:=\sqrt{{〈f,f〉}_{X^{o}}}$ is equivalent to ${\left\|f\right\|}_{X}$. Therefore, $\left(X,{\left\|f\right\|}_{X^{o}}\right)$ is a Hilbert space which is isomorphic to $\left(X,{\left\|f\right\|}_{X}\right)$. Now let $\xi_{l}:=D_{\Psi }D_{B}^{-1}\delta_{l}$, where *δ_l_* is the standard basis in $\ell^{2}$. This is a Hilbert space frame for *X*. Similarly, $\eta_{l}:=D_{\tilde{\Psi }}{\left(D_{B}^{⋆}\right)}^{-1}\delta_{l}$ is a Hilbert space frame for X′. As a consequence *X* and X′ are Hilbert spaces, but X=X′ and the inner products and the corresponding norms are changed, albeit equivalent to the original ones.

### 3.4. Stevenson frames

We consider the duality (H,H′) without using the Riesz isomorphism. In particular, we use the duality with respect to a second Hilbert space $H_{0}$.

Definition 3.1 ([17]). *A sequence* $\Psi ={\left(\psi_{k}\right)}_{k\in K}\subset H$ *is called a (Stevenson) frame for* H *if there exists constants* $0<A_{\Psi }\leq B_{\Psi }<\infty$ *such that*

(7) $$A_{\Psi }\cdot {\left\|f\right\|}_{H\prime }^{2}\leq {\left\|{〈f,\psi_{k}〉}_{H\prime ,H}\right\|}_{\ell^{2}}\leq B_{\Psi }\cdot {\left\|f\right\|}_{H\prime }^{2}\;\text{for}\;\text{all}\;f\in H\prime .$$

Different to the Gelfand frames setting, we do not assume density.

Typically, we consider Sobolev spaces and the *L* ^2^-inner product, which we can consider as co-orbit spaces with the sequence spaces $\ell_{w}^{2}$ varying *w*. Here, invertible operators between different spaces exist, see Section 3.3.2, and density is also given. In this article, we treat the most general setting.

In [17], the author states ‘We adapted the definition of a frame given in [39, Section 3] by identifying H with its dual H′ via the Riesz mapping’. Then, the following results are stated, also in [15], without proofs: The analysis operator $C_{\Psi }:H\prime \to \ell^{2}$, $C_{\Psi }(f)={\left(〈f,\psi_{k}〉\right)}_{k\in K}$ is bounded by (7), as is its adjoint $C_{\Psi }^{⋆}:\ell^{2}\to H$. It can be easily shown that $C_{\Psi }^{⋆}=D_{\Psi }$ is the synthesis operator with $D_{\Psi }c=\sum_{k\in K}c_{k}\psi_{k}$. Especially, one has

$$\ell^{2}=\text{ran}\left(C_{\Psi }\right)⊕\ker\left(D_{\Psi }\right).$$

Define the frame operator $S_{\Psi }=D_{\Psi }C_{\Psi }$. It is a mapping $S_{\Psi }:H\prime \to H$, which is boundedly invertible. We can show that the sequence $\tilde{\Psi }={\left(S_{\Psi }^{-1}\psi_{k}\right)}_{k\in K}$ is a (Stevenson) H-frame with bounds $\frac{1}{B_{\Psi }}$ and $\frac{1}{A_{\Psi }}$. Here, $C_{\tilde{\Psi }}=C_{\Psi }S_{\Psi }^{-1}$ and $D_{\tilde{\Psi }}=S_{\Psi }^{-1}D_{\Psi }$. Furthermore, it holds $S_{\tilde{\Psi }}=S_{\Psi }^{-1}$ and, therefore, $S_{\tilde{\Psi }}:H\to H\prime$.

We have the reconstructions

(8) $$f=D_{\Psi }C_{\tilde{\Psi }}h=\sum_{k\in K}{〈f,{\tilde{\psi }}_{k}〉}_{H,H\prime }\psi_{k},$$

and

(9) $$h=D_{\tilde{\Psi }}C_{\Psi }h=\sum_{k\in K}{〈h,\psi_{k}〉}_{H\prime ,H}{\tilde{\psi }}_{k},$$

for all f∈H and h∈H′.

The cross-Gramian matrix $G_{\Psi ,\tilde{\Psi }}=D_{\Psi }C_{\tilde{\Psi }}$ is the orthogonal projection on $\text{ran}\left(C_{\Psi }\right)$ and coincides with $G_{\tilde{\Psi },\Psi }$. Therefore, $\text{ran}\left(C_{\Psi }\right)=\text{ran}\left(C_{\tilde{\Psi }}\right)$.

In this article, we are revisiting those statements, make them slightly more general, in order to make sure that not using the Riesz isomorphism is possible.

### 3.5. An illustrative example

Let $\Omega \subset R^{n}$ be a sufficiently smooth, bounded domain. We consider a multiscale analysis, i.e., a dense, nested sequence of finite dimensional subspaces

$$V_{0}\subset V_{1}\subset \ldots \subset V_{j}\subset \ldots \subset L^{2}(\Omega ),$$

consisting of piecewise polynomial ansatz functions $V_{j}=\mathrm{span}\{\varphi_{j,k}:k\in \Delta_{j}\}$, such that $\dim V_{j}∼2^{jn}$ and

$$L^{2}(\Omega )=\bar{\underset{j\in N_{0}}{\cup }V_{j}},\;V_{0}=\underset{j\in N_{0}}{\cap }V_{j}.$$

One might think here of a multigrid decomposition of standard Lagrangian finite element spaces or of a sequence of spline spaces originating from dyadic subdivision.

Trial spaces *V_j_* which are used for the Galerkin method satisfy typically a *direct* or *Jackson* estimate. This means that

(10) $$\|v-P_{j}v{\|}_{L^{2}(\Omega )}\leq C_{J}2^{-jq}\|v{\|}_{H^{q}(\Omega )},\;v\in H^{q}(\Omega ),$$

holds for all 0≤q≤d uniformly in *j*. Here, $P_{j}:L^{2}(\Omega )\to V_{j}$ is the $L^{2}(\Omega )$-orthogonal projection onto the trial space *V_j_* and $H^{q}(\Omega )\subset L^{2}(\Omega ),q\geq 0$ denotes the Sobolev space of order *q*. The upper bound *d* > 0 refers in general to the maximum order of the polynomials which can be represented in *V_j_*, while the factor $2^{-j}$ refers to the mesh size of *V_j_*, i.e., the diameter of the finite elements, compare [26] for example.

Besides the Jackson type estimate (10), there also holds the *inverse* or *Bernstein* estimate

(11) $$\|P_{j}v{\|}_{H^{q}(\Omega )}\leq C_{B}2^{jq}\|P_{j}v{\|}_{L^{2}(\Omega )},\;v\in H^{q}(\Omega ),$$

for all 0≤q<γ, where the upper bound

$$\gamma :=\sup\{t\in R:V_{j}\subset H^{t}(\Omega )\}>0$$

refers to the regularity of the functions in the trial spaces *V_j_*. There holds γ=d−1/2 for trial functions based on cardinal B-splines, since they are globally $C^{d-1}$-smooth, and γ=3/2 for standard Lagrangian finite element shape functions, since they are only globally continuous.

A crucial requirement is the uniform frame stability of the systems under consideration, i.e., the existence of constants $A_{\Phi },B_{\Phi }>0$ such that

(12) $$A_{\Phi }\|P_{j}f{\|}_{L^{2}(\Omega )}^{2}\leq \sum_{k\in \Delta_{j}}|〈f,\varphi_{j,k}〉{|}^{2}\leq B_{\Phi }\|P_{j}f{\|}_{L^{2}(\Omega )}^{2}\;\text{for}\;\text{all}\;f\in L^{2}(\Omega )$$

holds uniformly for all *j*. This stability is satisfied for example by Lagrangian finite element basis functions defined on a multigrid hierarchy resulting from uniform refinement of a given coarse grid, see [26] for example. It is also satisfied by B-splines defined on a dyadic subdivision of the domain under consideration.

Having a multiscale analysis at hand, it can be used for telescoping a given function to account for the fact that Sobolev norms act different on different length scales. Namely, the interplay of (10) and (11) gives rise to the norm equivalence

(13) $$\|f{\|}_{{\tilde{H}}^{-q}(\Omega )}^{2}∼\sum_{j\in N_{0}}2^{-2jq}{\left\|(P_{j}-P_{j-1})f\right\|}_{L^{2}(\Omega )}^{2}$$

for all 0≤q<γ, where $P_{-1}:=0$ and ${\tilde{H}}^{-q}(\Omega ):=(H^{q}(\Omega ))\prime$ denotes the dual to $H^{q}(\Omega )$, see [40] for a proof.

In accordance with [15], using (12), we can estimate

$$\begin{array}{l} \sum_{j\in N_{0}}\sum_{k\in \Delta_{j}}2^{-2jq}|〈f,\varphi_{j,k}〉{|}^{2}\approx \sum_{j\in N_{0}}2^{-2jq}||P_{j}f{\|}_{L^{2}(\Omega )}^{2}\\ =\sum_{j\in N_{0}}2^{-2jq}\sum_{\ell =0}^{j}\|(P_{\ell }-P_{\ell -1})f{\|}_{L^{2}(\Omega )}^{2}\\ =\sum_{\ell \in N_{0}}\|(P_{\ell }-P_{\ell -1})f{\|}_{L^{2}(\Omega )}^{2}\sum_{j=\ell }^{\infty }2^{-2jq}. \end{array}$$

The latter sum converges provided that *q* > 0 and we arrive at

$$\sum_{j\in N_{0}}\sum_{k\in \Delta_{j}}2^{-2jq}|〈f,\varphi_{j,k}〉{|}^{2}\approx \sum_{\ell \in N_{0}}2^{-2\ell q}\|(P_{\ell }-P_{\ell -1})f{\|}_{L^{2}(\Omega )}^{2}.$$

In view of the norm equivalence (13), we have thus proven that there exist constants $A_{\Phi },B_{\Phi }>0$ such that

(14) $$A_{\Phi }\|f{\|}_{{\tilde{H}}^{-q}(\Omega )}^{2}\leq \sum_{j\in N_{0}}\sum_{k\in \Delta_{j}}2^{-2jq}|〈f,\varphi_{j,k}〉{|}^{2}\leq B_{\Phi }\|f{\|}_{{\tilde{H}}^{-q}(\Omega )}^{2}$$

for all 0<q<γ. Therefore, in accordance with Definition 3.1, the collection

(15) $$\Phi =\{2^{-jq}\varphi_{j,k}:k\in \Delta_{j},\;j\in N_{0}\}$$

defines a Stevenson frame for $H=H^{q}(\Omega )$, where $H\prime ={\tilde{H}}^{-q}(\Omega )$ with duality related to $H_{0}=L^{2}(\Omega )$. Notice that this frame underlies the construction of the so-called BPX preconditioner, see e.g., [40–42]. Especially, by removing all basis functions which are associated with boundary nodes, one gets a Stevenson frame for $H=H_{0}^{1}(\Omega )$, as required for the Galerkin discretization of elliptic partial differential equations, compare Section 2.

We like to emphasize that the collection (15) does not define a Gelfand frame, since (14) does not hold in $H_{0}=L^{2}(\Omega )$, i.e., for *q* = 0. Hence, the concept of Stevenson frames seems to be more flexible than the concept of Gelfand frames.

### 3.6. Operator representation in frame coordinates

For orthonormal sequences, it is well known that operators can be uniquely described by a matrix representation [43]. The same can be constructed with frames and their duals, see [18, 19].

Let $\Psi =(\psi_{k})$ be a frame in $H_{1}$ with bounds $A_{\Psi },B_{\Psi }>0$, and let $\Phi =(\phi_{k})$ be a frame in $H_{2}$ with $A_{\Phi },B_{\Phi }>0$.

1. Let $O:H_{1}\to H_{2}$ be a bounded, linear operator. Thus, the infinite matrix

$${\left(M^{\left(\Phi ,\Psi \right)}\left(O\right)\right)}_{m,n}=〈O\psi_{n},\phi_{m}〉$$

defines a bounded operator from $\ell^{2}$ to $\ell^{2}$ with ${\left\|M\right\|}_{\ell^{2}\to \ell^{2}}\leq \sqrt{B_{\Phi }\cdot B_{\Psi }}\cdot {\left\|O\right\|}_{H_{1}\to H_{2}}$. As an operator $\ell^{2}\to \ell^{2}$, we have

$$M^{\left(\Phi ,\Psi \right)}\left(O\right)=C_{\Phi }{}^{\circ}O{}^{\circ}D_{\Psi }.$$

2. On the other hand, let *M* be an infinite matrix defining a bounded operator from $\ell^{2}$ to $\ell^{2},{\left(Mc\right)}_{i}=\sum_{k}M_{i,k}c_{k}$. Then, the operator $O^{\left(\Phi ,\Psi \right)}$ defined by
  $$\left(O^{\left(\Phi ,\Psi \right)}\left(M\right)\right)h=\sum_{k}\left(\sum_{j}M_{k,j}〈h,\psi_{j}〉\right)\phi_{k}\;\text{for}\;\text{all}\;h\in H_{1}$$

is a bounded operator from $H_{1}$ to $H_{2}$ with

$${\left\|O^{\left(\Phi ,\Psi \right)}\left(M\right)\right\|}_{H_{1}\to H_{2}}\leq \sqrt{B_{\Phi }\cdot B_{\Psi }}{\left\|M\right\|}_{\ell^{2}\to \ell^{2}}$$

and

$$O^{\left(\Phi ,\Psi \right)}(M)=D_{\Phi }{}^{\circ}M{}^{\circ}C_{\Psi }=\sum_{k}\sum_{j}M_{k,j}\cdot \phi_{k}{⊗}_{i}\psi_{j}.$$

Please note that there is a classification of matrices that are bounded operators from $\ell^{2}$ to $\ell^{2}$ [44].

If we start out with frames, more properties can be proved [18]: Let $\Psi =(\psi_{k})$ be a frame in $H_{1}$ with bounds $A_{\Psi },B_{\Psi }>0,\Phi =(\phi_{k})$ in $H_{2}$ with $A_{\Phi },B_{\Phi }>0$.

1. It holds

$$\left(O^{\left(\Phi ,\Psi \right)}{}^{\circ}M^{\left(\tilde{\Phi },\tilde{\Psi }\right)}\right)={\text{id}}_{B(H_{1},H_{2})}=\left(O^{\left(\tilde{\Phi },\tilde{\Psi }\right)}{}^{\circ}M^{\left(\Phi ,\Psi \right)}\right).$$

Therefore, for all $O\in B(H_{1},H_{2})$:

$$O=\sum_{k,j}〈O{\tilde{\psi }}_{j},{\tilde{\phi }}_{k}〉\phi_{k}{⊗}_{i}\psi_{j}.$$

2. $M^{\left(\Phi ,\Psi \right)}$ is injective and $O^{\left(\Phi ,\Psi \right)}$ is surjective.

3. If $H_{1}=H_{2}$, then $O^{\left(\Psi ,\tilde{\Psi }\right)}({\text{id}}_{\ell^{2}})={\text{id}}_{H_{1}}$.

4. Let $\Xi =(\xi_{k})$ be any frame in $H_{3}$, and $O:H_{3}\to H_{2}$ and $P:H_{1}\to H_{3}$. Then, it holds

$$M^{\left(\Phi ,\Psi \right)}\left(O{}^{\circ}P\right)=\left(M^{\left(\Phi ,\Xi \right)}\left(O\right)\cdot M^{\left(\tilde{\Xi },\Psi \right)}\left(P\right)\right).$$

Note that, in the Hilbert space of Hilbert–Schmidt operators, the tensor product $\Psi ⊗\Phi :={\left\{\psi_{k}⊗\psi_{l}\right\}}_{(k,l)\in K\times K}$ is a Bessel sequence/frame sequence/Riesz sequence, if the starting sequences Ψ and Φ are [45], with $M^{\left(\Phi ,\Psi \right)}$ being the analysis and $O^{\left(\Phi ,\Psi \right)}$ being the synthesis operator. This relation is even an equivalence [46].

For the invertibility, it can be shown [20, 47]: If and only if *O* is bijective, then $M=M^{\left(\Phi ,\Psi \right)}(O)$ is bijective as operator from $\text{ran}\left(C_{\Psi }\right)$ onto $\text{ran}\left(C_{\Phi }\right)$. In this case, one has

$$M^{†}=M^{\left(\tilde{\Psi },\tilde{\Phi }\right)}(O^{-1})=G_{\tilde{\Psi },\tilde{\Phi }}{}^{\circ}M^{\left(\Phi ,\Psi \right)}\left(O^{-1}\right)G_{\tilde{\Psi },\tilde{\Phi }}=M^{\left(\Psi ,\Phi \right)}\left(S_{\Psi }^{-1}O^{-1}S_{\Phi }^{-1}\right).$$

If we have an operator equation Ou=b, we use

$$Ou=b\Leftrightarrow \sum_{k}〈u,{\tilde{\psi }}_{k}〉O\psi_{k}=b,$$

which implies

$$\sum_{k}〈u,{\tilde{\psi }}_{k}〉〈O\psi_{k},\psi_{l}〉=〈b,\psi_{l}〉$$

for all l∈K. Setting $M=M^{\left(\Psi ,\Psi \right)}(O)$, $\vec{u}=C_{\tilde{\Psi }}u$ and $\vec{b}=C_{\Psi }b$, we thus have

$$Ou=b\Leftrightarrow M\vec{u}=\vec{b}.$$

Note that, for numerical computations, see e.g. [17, 21], the system of linear equations $M\vec{u}=\vec{b}$ is solved. Then, $u=D_{\Psi }\vec{u}$ is the solution to Ou=b, avoiding the numerically expensive calculation of a dual frame [48–50]. If the frame is redundant, then *u_k_* can be different to $〈u,{\tilde{\psi }}_{k}〉$. If a Tychonov regularization is used, we obtain $u_{k}=〈u,{\tilde{\psi }}_{k}〉$ by [51, Prop. 5.1.4].

## 4. Stevenson frames revisited

As some of the references dealing with Stevenson frames used an unlucky formulation, when stating if or if not the Riesz isomorphism is used, see e.g. [17, 21], the authors decided to check everything again, and pay particular attention to the avoidance of the Riesz isomorphism, i.e., to not use H≅H′.

To *not* use the Riesz isomorphism in a treatment of Hilbert spaces is mind-boggling, so we decided to use Banach spaces, to be sure to avoid all pitfalls. (Note, however, that the Riesz isomorphism will be used on the sequence space $\ell^{2}$.) In particular, this is a generalization of the original definition. The used spaces are necessarily isomorphic to Hilbert spaces, but not Hilbert spaces *per se*.

### 4.1. Stevenson Banach frames

We start out with a generalized definition. (We will show that this is isomorphic, but *not* identical to the original definition.)

Definition 4.1. *Let (X,X′) be a dual pair of reflexive Banach spaces. Let $\Psi ={\left(\psi_{k}\right)}_{k\in K}\subset X$. It is called a Stevenson Banach frame for X, if there exist bounds $0<A_{\Psi }\leq B_{\Psi }<\infty$ such that*

$$A_{\Psi }{\left\|f\right\|}_{X\prime }^{2}\leq {\left\|{〈f,\psi_{k}〉}_{X\prime ,X}\right\|}_{\ell^{2}}\leq B_{\Psi }{\left\|f\right\|}_{X\prime }^{2}\;\text{for}\;\text{all}\;f\in X\prime .$$

The analysis operator

$$C_{\Psi }:X\prime \to \ell^{2},\;C_{\Psi }(f)={\left({〈f,\psi_{k}〉}_{X\prime ,X}\right)}_{k\in K}$$

is bounded by $\sqrt{B_{\Psi }}$ by definition. (Note that we use here the notation which is more common for Banach spaces [24].) As a consequence of the open mapping theorem, $C_{\Psi }$ is one-to-one and has closed range.

For $d=(d_{k})\in \ell^{2}(K)$ with finitely many nonzero entries, i.e., $d\in c^{00}$, consider

$${〈C_{\Psi }f,d〉}_{\ell^{2}}=\sum_{k\in K}{〈f,\psi_{k}〉}_{X\prime ,X}d_{k}={〈f,\sum_{k\in K}d_{k}\psi_{k}〉}_{X\prime ,X}.$$

By using a standard density argument and the reflexivity, it can easily be shown that $C_{\Psi }^{⋆}=D_{\Psi }$, where $D_{\Psi }:\ell^{2}\to X$ is the synthesis operator with $D_{\Psi }c=\sum_{k\in K}c_{k}\psi_{k}$. The bound of $D_{\Psi }$ is also $\sqrt{B_{\Psi }}$. The sum converges unconditionally. Indeed, consider $c\in \ell^{2}$. Then, let $K_{0}\subset K$ be a finite set, such that

$$\sum_{k\notin K_{0}}{\left|c_{k}\right|}^{2}<\epsilon \prime :=\frac{\epsilon }{\sqrt{B_{\Psi }}}.$$

For another finite index set $K_{1}⊃K_{0}$, we thus find

$${\left\|\sum_{k\notin K}c_{k}\psi_{k}-\sum_{k\notin K_{1}}c_{k}\psi_{k}\right\|}_{H}={\left\|D_{\Psi }\left(c-c\cdot \chi_{K_{1}}\right)\right\|}_{H}<\sqrt{B_{\Psi }}\epsilon \prime =\epsilon .$$

Hence, by e.g. [28, IV.5.1] and the fact that $\ell^{2}$ is a Hilbert space, we deduce

(16) $$\ell^{2}=\text{ran}\left(C_{\Psi }\right)⊕\ker\left(D_{\Psi }\right).$$

We define the frame operator $S_{\Psi }=D_{\Psi }C_{\Psi }$, which is a mapping $S_{\Psi }:X\prime \to X$. In particular, the operator $S_{\Psi }$ is self-adjoint. By definition of $S_{\Psi }$, it follows that

(17) $${〈S_{\Psi }f,g〉}_{X,X\prime }\leq {〈C_{\Psi }f,C_{\Psi }g〉}_{\ell^{2}},\leq B_{\Psi }\cdot {\left\|f\right\|}_{X\prime }\cdot {\left\|g\right\|}_{X\prime }.$$

Hence, $S_{\Psi }$ is bounded with bound $B_{\Psi }$. Furthermore, we have

(18) $${〈S_{\Psi }f,f〉}_{X,X\prime }={〈C_{\Psi }^{⋆}C_{\Psi }f,f〉}_{X,X\prime }={〈C_{\Psi }f,C_{\Psi }f〉}_{\ell^{2}}={\left\|C_{\Psi }f\right\|}_{\ell^{2}}^{2}\geq A_{\Psi }\cdot {\left\|f\right\|}_{X\prime }^{2},$$

which implies that $S_{\Psi }$ is one-to-one and positive. By [28, IV.5.1], this also means that $S_{\Psi }^{⋆}=S_{\Psi }$ has dense range. $S_{\Psi }$ also has a bounded inverse since

$${\left\|S_{\Psi }f\right\|}_{X}=\underset{\begin{array}{c} {\left\|g\right\|}_{X\prime }=1\\ g\in X\prime \end{array}}{\sup}{〈S_{\Psi }f,g〉}_{X,X\prime }\geq {〈S_{\Psi }f,\frac{f}{{\left\|f\right\|}_{X\prime }}〉}_{X,X\prime }\geq A_{\Psi }\cdot {\left\|f\right\|}_{X\prime }.$$

Therefore, it has closed range [52, Theorem XI.2.1]. Consequently, $S_{\Psi }$ is onto and bijective with

$$A_{\Psi }{\left\|f\right\|}_{X\prime }\leq {\left\|S_{\Psi }f\right\|}_{X}\leq B_{\Psi }{\left\|f\right\|}_{X\prime }.$$

Thus, $S_{\Psi }^{-1}$ is also self-adjoint, and

(19) $$\frac{1}{B_{\Psi }}{\left\|g\right\|}_{X}\leq {\left\|S_{\Psi }^{-1}g\right\|}_{X\prime }\leq \frac{1}{A_{\Psi }}{\left\|g\right\|}_{X}.$$

Theorem 4.1. The sequence $\tilde{\Psi }={\left({\tilde{\psi }}_{k}\right)}_{k\in K}:={\left(S_{\Psi }^{-1}\psi_{k}\right)}_{k\in K}\subset X\prime$ is a Stevenson Banach frame for X′ with bounds $\frac{1}{B_{\Psi }}$ and $\frac{1}{A_{\Psi }}$. The range of its analysis operator coincides with the one of the primal frame, i.e., $\text{ran}\left(C_{\Psi }\right)=\text{ran}\left(C_{\tilde{\Psi }}\right)$. The related operators are $C_{\tilde{\Psi }}=C_{\Psi }S_{\Psi }^{-1},D_{\tilde{\Psi }}=S_{\Psi }^{-1}D_{\Psi }$ and $S_{\tilde{\Psi }}=S_{\Psi }^{-1}$. For f∈X and g∈X′, we have the reconstructions

$$f=\sum_{k\in K}{〈f,{\tilde{\psi }}_{k}〉}_{X,X\prime }\psi_{k}\;\text{and}\;g=\sum_{k\in K}{〈g,\psi_{k}〉}_{X\prime ,X}{\tilde{\psi }}_{k}.$$

Proof. It obviously holds $S_{\Psi }^{-1}\psi_{k}\in X\prime$. Moreover, we have on the one hand

$$\begin{array}{c} \sum_{k\in K}{\left|{〈f,{\tilde{\psi }}_{k}〉}_{X,X\prime }\right|}^{2}=\sum_{k\in K}{\left|{〈f,S_{\Psi }^{-1}\psi_{k}〉}_{X,X\prime }\right|}^{2}=\sum_{k\in K}{\left|{〈S_{\Psi }^{-1}f,\psi_{k}〉}_{X\prime ,X}\right|}^{2}\\ \leq B_{\Psi }{\left\|S_{\Psi }^{-1}f\right\|}_{X\prime }^{2}\leq \frac{B_{\Psi }}{A_{\Psi }^{2}}{\left\|f\right\|}_{X}^{2} \end{array}$$

and on the other hand

$$\sum_{k\in K}{\left|{〈f,{\tilde{\psi }}_{k}〉}_{X,X\prime }\right|}^{2}\geq A_{\Psi }{\left\|S_{\Psi }^{-1}f\right\|}_{X\prime }^{2}\geq \frac{A_{\Psi }}{B_{\Psi }^{2}}{\left\|f\right\|}_{X\prime }^{2}.$$

Hence, $\tilde{\Psi }$ is an X′-frame. By employing the invertibility of $S_{\Psi }$ for $g=S_{\Psi }^{-1}f$, we get

$${〈f,S_{\Psi }^{-1}\psi_{k}〉}_{X,X\prime }={〈S_{\Psi }g,S_{\Psi }^{-1}\psi_{k}〉}_{X,X\prime }={〈g,S_{\Psi }S_{\Psi }^{-1}\psi_{k}〉}_{X\prime ,X}={〈g,\psi_{k}〉}_{X\prime ,X}.$$

This implies $\text{ran}\left(C_{\Psi }\right)=\text{ran}\left(C_{\tilde{\Psi }}\right)$, where $C_{\tilde{\Psi }}=C_{\Psi }S_{\Psi }^{-1}$ and $D_{\tilde{\Psi }}=S_{\Psi }^{-1}D_{\Psi }$. Furthermore, $S_{\tilde{\Psi }}=S_{\Psi }^{-1}:X\to X\prime$, because it holds

$$\begin{array}{l} S_{\tilde{\Psi }}f=\sum_{k}{〈f,S_{\Psi }^{-1}\psi_{k}〉}_{X,X\prime }S_{\Psi }^{-1}\psi_{k}\\ =S_{\Psi }^{-1}\sum_{k}{〈S_{\Psi }^{-1}f,\psi_{k}〉}_{X,X\prime }\psi_{k}=S_{\Psi }^{-1}S_{\Psi }S_{\Psi }^{-1}f=S_{\Psi }^{-1}f \end{array}$$

for all f∈X.

Finally, we have the reconstructions

$$f=D_{\Psi }C_{\Psi }S_{\Psi }^{-1}f=D_{\Psi }C_{\tilde{\Psi }}f=\sum_{k\in K}{〈f,{\tilde{\psi }}_{k}〉}_{X,X\prime }\psi_{k}$$

for all f∈X and

$$g=S_{\Psi }^{-1}D_{\Psi }C_{\Psi }=D_{\tilde{\Psi }}C_{\Psi }g=\sum_{k\in K}{〈h,\psi_{k}〉}_{X\prime ,X}{\tilde{\psi }}_{k}$$

for all g∈X′.

As

$${〈S_{\Psi }^{-1}x,x〉}_{X\prime ,X}\leq {\left\|S_{\Psi }^{-1}x\right\|}_{X\prime }{\left\|x\right\|}_{X}\leq \frac{1}{A_{\Psi }}{\left\|x\right\|}_{X}^{2},$$

we have the sharper upper bound. On the other hand, since $〈S_{\Psi }^{-1}\cdot ,\cdot 〉$ defines a positive sesquilinear form, the Cauchy–Schwarz inequality implies

$${\left|{〈S_{\Psi }^{-1}x,y〉}_{X\prime ,X}\right|}^{2}\leq {〈S_{\Psi }^{-1}x,x〉}_{X\prime ,X}{〈S_{\Psi }^{-1}y,y〉}_{X\prime ,X}.$$

Thus, with $x=S_{\Psi }u$, there holds

$${\left|{〈u,y〉}_{X\prime ,X}\right|}^{2}\leq {〈u,S_{\Psi }u〉}_{X\prime ,X}{〈S_{\Psi }^{-1}y,y〉}_{X\prime ,X}$$

and consequently

$${\left\|y\right\|}_{X}^{2}=\underset{\begin{array}{c} {\left\|u\right\|}_{X\prime }=1\\ u\in X\prime \end{array}}{\sup}{\left|{〈u,y〉}_{X\prime ,X}\right|}^{2}\leq B_{\Psi }{〈S_{\Psi }^{-1}y,y〉}_{X\prime ,X}.$$

So, the sharper bounds $\frac{1}{B_{\Psi }}$ and $\frac{1}{A_{\Psi }}$ follow. □

The fact that $\text{ran}\left(C_{\Psi }\right)=\text{ran}\left(C_{\tilde{\Psi }}\right)$ is very different to the Gelfand frame setting, where the ranges ran(CΨ|X′)=ran(CΨ˜|X) even live in different sequence spaces.

Theorem 4.2. *The cross-Gramian matrix* $G_{\Psi ,\tilde{\Psi }}=C_{\Psi }D_{\tilde{\Psi }}$ *is the orthogonal projection on* $\text{ran}\left(C_{\Psi }\right)$ *and coincides with* $G_{\tilde{\Psi },\Psi }$.

Proof. We have that the cross-Gramian matrix of a frame and its dual is a projection:

$${\left(G_{\Psi ,\tilde{\Psi }}\right)}^{2}=C_{\Psi }D_{\tilde{\Psi }}C_{\Psi }D_{\tilde{\Psi }}=C_{\Psi }D_{\tilde{\Psi }}.=G_{\Psi ,\tilde{\Psi }}.$$

Next, it holds

$$G_{\Psi ,\tilde{\Psi }}^{⋆}={\left(C_{\Psi }D_{\tilde{\Psi }}\right)}^{⋆}=C_{\tilde{\Psi }}D_{\Psi }=G_{\tilde{\Psi },\Psi }.$$

In addition, since

$${\left(G_{\Psi ,\tilde{\Psi }}\right)}_{k,l}={〈{\tilde{\psi }}_{l},\psi_{k}〉}_{X\prime ,X}={〈S_{\Psi }^{-1}\psi_{l},\psi_{k}〉}_{X\prime ,X}={〈\psi_{l},S_{\Psi }^{-1}\psi_{k}〉}_{X,X\prime }={\left(G_{\tilde{\Psi },\Psi }\right)}_{k,l},$$

we conclude $G_{\Psi ,\tilde{\Psi }}=G_{\tilde{\Psi },\Psi }$. Thus, $G_{\Psi ,\tilde{\Psi }}$ is self-adjoint.□

Theorem 4.3. *The collection* Ψ *is a Stevenson Banach frame for X with bounds* $A_{\Psi }$ *and* $B_{\Psi }$ *if and only if*

$$\frac{1}{B_{\Psi }}{\left\|f\right\|}_{X}\leq \underset{d\in \ell^{2},\;D_{\Psi }d=f}{\inf}{\left\|d\right\|}_{\ell^{2}}\leq \frac{1}{A_{\Psi }}{\left\|f\right\|}_{X}.$$

*In particular, for any f∈X with $f=\sum_{k\in K}d_{k}\psi_{k}$ and $d=(d_{k})\in \ell^{2}$, we have ${\left\|d\right\|}_{\ell^{2}}\geq {\left\|C_{\tilde{\Psi }}f\right\|}_{\ell^{2}}$.*

Proof. Given

$$f=\sum_{k\in K}d_{k}\psi_{k}\in X,$$

we have the representation

$$f=\sum_{k\in K}{〈f,{\tilde{\psi }}_{k}〉}_{X,X\prime }\psi_{k}.$$

Hence,

$$\left(d_{k}-{〈f,{\tilde{\psi }}_{k}〉}_{X,X\prime }\right)\in \ker\left(D_{\Psi }\right).$$

By (16) and Theorem 4.1, there follows ${\left\|d\right\|}_{\ell^{2}}\geq {\left\|C_{\tilde{\Psi }}f\right\|}_{\ell^{2}}$.□

Consequently, a Stevenson frame is a Riesz basis for *X* if and only if $D_{\Psi }$ is one-to-one.

### 4.2. Is 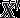 X′ a Hilbert space?

Set ${〈u,v〉}_{X\prime_{H}}:={〈u,S_{\Psi }v〉}_{X\prime ,X}$. This is, trivially, a symmetric and positive bilinear form by above and, therefore, an inner product on X′. Hence, X′ is a pre-Hilbert space with this inner product. By (17) and (18), the corresponding norm is equivalent to the original one as $\sqrt{A_{\Psi }}{\left\|f\right\|}_{X\prime }\leq {\left\|f\right\|}_{X\prime_{H}}\leq \sqrt{B_{\Psi }}{\left\|f\right\|}_{X\prime }$. Thus, (X′,〈·,·〉) is a Hilbert space.

Note that, in particular for numerics, it is sometimes not enough to consider equivalent norms. While well-posed problems stay well-posed for equivalent norms, this becomes important for concrete implementations, as things like condition numbers, constants in convergence rates, etc. are considered.

From a frame theory perspective, switching to an equivalent norm can destroy or create tightness, in particular, the switch from one norm to the other changes the frame bound ratio $\frac{B_{\Psi }}{A_{\Psi }}$. We refer the reader to, e.g., weighted and controlled frames [37], which are under very mild conditions equivalent to classical Hilbert frames. Nonetheless, they have applications for example in the implementation of wavelets on the sphere [53, 54], and nowadays become important for the scaling of frames [55, 56]. As a trivial example, look at $\Psi :=\left\{e_{1},e_{1},e_{2},e_{2},e_{3},e_{3},\ldots \right\}$, where $E={\left\{e_{i}\right\}}_{i\in N}$ is an orthonormal basis for H. Then, Ψ is a tight frame with $A_{\Psi }=2$. Looking at the reweighted version $\Phi :=\left\{2e_{1},2e_{1},e_{2}/2,e_{2}/2,2e_{3},2e_{3},\ldots \right\}$, we loose tightness, since this frame has bounds *A* = 1 and *B* = 4. Note that there exists an invertible bounded operator that maps the single elements from Ψ into Φ, i.e., they are equivalent sequences [57].

Also note that, if it does not make sense to assume that X⊂X′, then Ψ cannot be a Hilbert space frame *per se*. This can only be true for the subsequence of *X*′ the Stevenson frame $\Psi \prime :=(\psi \prime_{k})=(I\psi_{k})$, where *I* is an isomorphism from X′ to *X*, for example, choosing $I=S_{\Psi }^{-1}$. In this case, the frame bounds are preserved, but the roles of primal and dual frames interchange.

This especially means that, if the frame bound ratio is important, distinguishing $\ell^{2}$-Banach frames from Hilbert frames is necessary, especially if concrete examples for *X* and X′ are used, where an identification is not possible, i.e., X=X′. As such, Definition 4.1 is, of course, equivalent to the standard frame definition for Hilbert spaces, but *the frame bound ratio changes*.

We like to remark that, by using the dual frame, one can also conclude that *X* itself is a Hilbert space.

### 4.3. Matrix representation

Let us also revisit the statements about the matrix representation of operators [15, 17]. To this end, let Ψ be Stevenson Banach frame for *X*.

Let us now consider an operator O:X→X′ and define

$${\left(M^{\left(\Psi \right)}\left(O\right)\right)}_{m,n}={〈O\psi_{n},\psi_{m}〉}_{X\prime ,X}.$$

Then,

$$M^{\left(\Psi \right)}\left(O\right)=C_{\Psi }OD_{\Psi }$$,

which implies

$${\left\|M^{\left(\Psi \right)}\left(O\right)\right\|}_{\ell^{2}\to \ell^{2}}\leq B_{\Psi }{\left\|O\right\|}_{X\to X\prime }.$$

(As in Section 3.6, we could consider different sequences, and the arguments would still work, but following the argument in the Introduction and for easy reading we will not.)

For an invertible operator *O*, we have

$$M^{\left(\tilde{\Psi }\right)}(O^{-1})M^{\left(\Psi \right)}(O)=C_{\tilde{\Psi }}O^{-1}D_{\tilde{\Psi }}C_{\Psi }OD_{\Psi }=G_{\tilde{\Psi },\Psi }.$$

(For the analog result in the Hilbert frame case, see [20, 47].) Equivalently,

$$M^{\left(\Psi \right)}(O)M^{\left(\tilde{\Psi }\right)}(O^{-1})=G_{\tilde{\Psi },\Psi }.$$

Therefore, as $G_{\tilde{\Psi },\Psi }$ is the orthogonal projection on $\text{ran}\left(C_{\Psi }\right)$ the operator $M^{\left(\Psi \right)}{\left(O\right)}_{{|}_{\text{ran}\left(C_{\Psi }\right)}}$ is boundedly invertible, as

$${\left\|M^{\left(\Psi \right)}(O)\right\|}_{\text{ran}\left(C_{\Psi }\right)\to \text{ran}\left(C_{\Psi }\right)}\geq A_{\Psi }{\left\|O^{-1}\right\|}_{X\prime \to X}^{-1}.$$

Furthermore,

$$\ker\left(M^{\left(\tilde{\Psi }\right)}(O)\right)=\ker\left(D_{\Psi }\right).$$

If *O* is symmetric, then $M^{\left(\Psi \right)}\left(O\right)$ is symmetric. If *O* is nonnegative, so is $M^{\left(\Psi \right)}\left(O\right)$. In particular, we have now have settled all statements in [15, 17].
